# Higher Framingham steatosis index is associated with prevalent breast cancer in women: cross-sectional evidence from NHANES 1999–2018 and an exploratory hospital-based dataset

**DOI:** 10.3389/fonc.2026.1849341

**Published:** 2026-07-08

**Authors:** Shuling Tang, Yong Mo, Tiansheng Su, Guangxiang Huang, Jiachao Lu, Jianbin Bi, Hui Li, Ligen Mo, Jun Yan

**Affiliations:** 1 Department of Neurosurgery, Guangxi Medical University Cancer Hospital, Nanning, China; 2 Department of Neurosurgery, The First Affiliated Hospital of Guangxi Medical University, Nanning, China

**Keywords:** breast cancer, cross-sectional study, Framingham steatosis index, hepatic steatosis, metabolic dysfunction, NHANES

## Abstract

**Background:**

Metabolic dysfunction and hepatic steatosis-related phenotypes have increasingly been linked to extrahepatic malignancies, including breast cancer. The association between the Framingham Steatosis Index (FSI), a composite index incorporating age and multiple metabolic components, and prevalent breast cancer in women has not been well characterized.

**Methods:**

We conducted a cross-sectional analysis of female participants in the National Health and Nutrition Examination Survey (NHANES) 1999–2018. Multivariable logistic regression models were used to evaluate the association between FSI and prevalent breast cancer, with FSI analyzed as both a continuous variable and by quartiles. Restricted cubic spline analysis and generalized additive modeling were applied to assess potential non-linearity, and two-piecewise logistic regression was used to explore a possible threshold effect. Component-level and AUC analyses compared FSI with its individual components. An exploratory hospital-based supportive dataset was analyzed to assess whether the continuous association showed a similar direction in a different clinical setting.

**Results:**

Among 21,042 women in NHANES, 531 (2.5%) reported a history of breast cancer. In the fully adjusted model, each 1-unit increase in FSI was associated with higher odds of prevalent breast cancer (OR 1.10, 95% CI 1.05–1.16). After additional adjustment for age, the association was substantially attenuated and no longer statistically significant (OR 1.02, 95% CI 0.96–1.07). Higher FSI quartiles were also associated with greater odds of prevalent breast cancer. Restricted cubic spline analysis suggested a non-linear association. In component-level analyses, age showed the strongest association and discriminatory performance among the FSI components, whereas adding FSI to the base model only modestly increased the AUC. In the exploratory hospital-based dataset, FSI was positively associated with breast cancer case status when modeled continuously, but the quartile-based dose-response pattern was not reproduced after full adjustment.

**Conclusions:**

In the primary NHANES analysis, higher FSI was associated with prevalent breast cancer status in women; however, this association was substantially attenuated after additional adjustment for age. FSI should therefore be interpreted as a composite marker of metabolic-hepatic burden rather than as an age-independent hepatic steatosis effect or causal biomarker.

## Introduction

Breast cancer remains the most frequently diagnosed malignancy in women worldwide and continues to impose a substantial public health burden despite major advances in screening, diagnosis, and treatment (1, 2). In addition to established reproductive, hormonal, genetic, and lifestyle-related determinants, increasing attention has been directed toward metabolic abnormalities in breast cancer epidemiology. Metabolic syndrome and related phenotypes, including obesity, insulin resistance, dyslipidemia, hypertension, and diabetes, have been associated with breast cancer risk and mortality, supporting a broader role for systemic metabolic dysregulation in breast cancer biology (3–5).

Hepatic steatosis is an important manifestation of metabolic dysfunction. Non-alcoholic fatty liver disease (NAFLD), now increasingly referred to as metabolic dysfunction-associated steatotic liver disease (MASLD) under the updated nomenclature, is closely linked to obesity, insulin resistance, dyslipidemia, and other cardiometabolic abnormalities (6). MASLD has also been associated with extrahepatic malignancies, including breast cancer (7–11). However, because hepatic steatosis-related phenotypes commonly coexist with multiple metabolic risk factors, their association with breast cancer should be interpreted as reflecting broader metabolic-hepatic burden rather than an isolated hepatic steatosis effect.

The Framingham Steatosis Index (FSI) is a validated and readily applicable non-invasive surrogate marker developed to identify hepatic steatosis in general populations (12, 13). It incorporates age, sex, body mass index, triglycerides, hypertension, diabetes, and the alanine aminotransferase/aspartate aminotransferase ratio, thereby capturing several interrelated demographic and metabolic components rather than a single abnormality (12, 13). Accordingly, in the context of cancer epidemiology, FSI should be interpreted as a composite indicator of hepatic steatosis-related metabolic dysfunction, not as a direct diagnostic measure of fatty liver or as an index independent of its individual components. Another non-invasive indicator, the Fatty Liver Index, has previously been associated with breast cancer in a nationwide population-based study, but whether FSI is associated with prevalent breast cancer in women has not been systematically evaluated (9).

Using data from the National Health and Nutrition Examination Survey (NHANES) 1999–2018, we therefore examined the association between FSI and prevalent breast cancer in adult women. We hypothesized that higher FSI would be associated with higher odds of prevalent breast cancer, while recognizing that the cross-sectional design precludes causal or temporal inference. We further assessed the shape of this association using non-linear modeling and conducted sensitivity analyses related to age and individual FSI components. In addition, we included an exploratory hospital-based supportive dataset from China to evaluate whether the continuous association showed a similar direction in a different clinical setting, rather than to provide confirmatory external validation.

## Materials and methods

### Study participants

This cross-sectional study used publicly available NHANES data from 1999 to 2018, which represent the non-institutionalized U.S. population. All procedures were approved by the National Center for Health Statistics (NCHS) Research Ethics Review Board, and participant consent was obtained.

**Figure 1:**
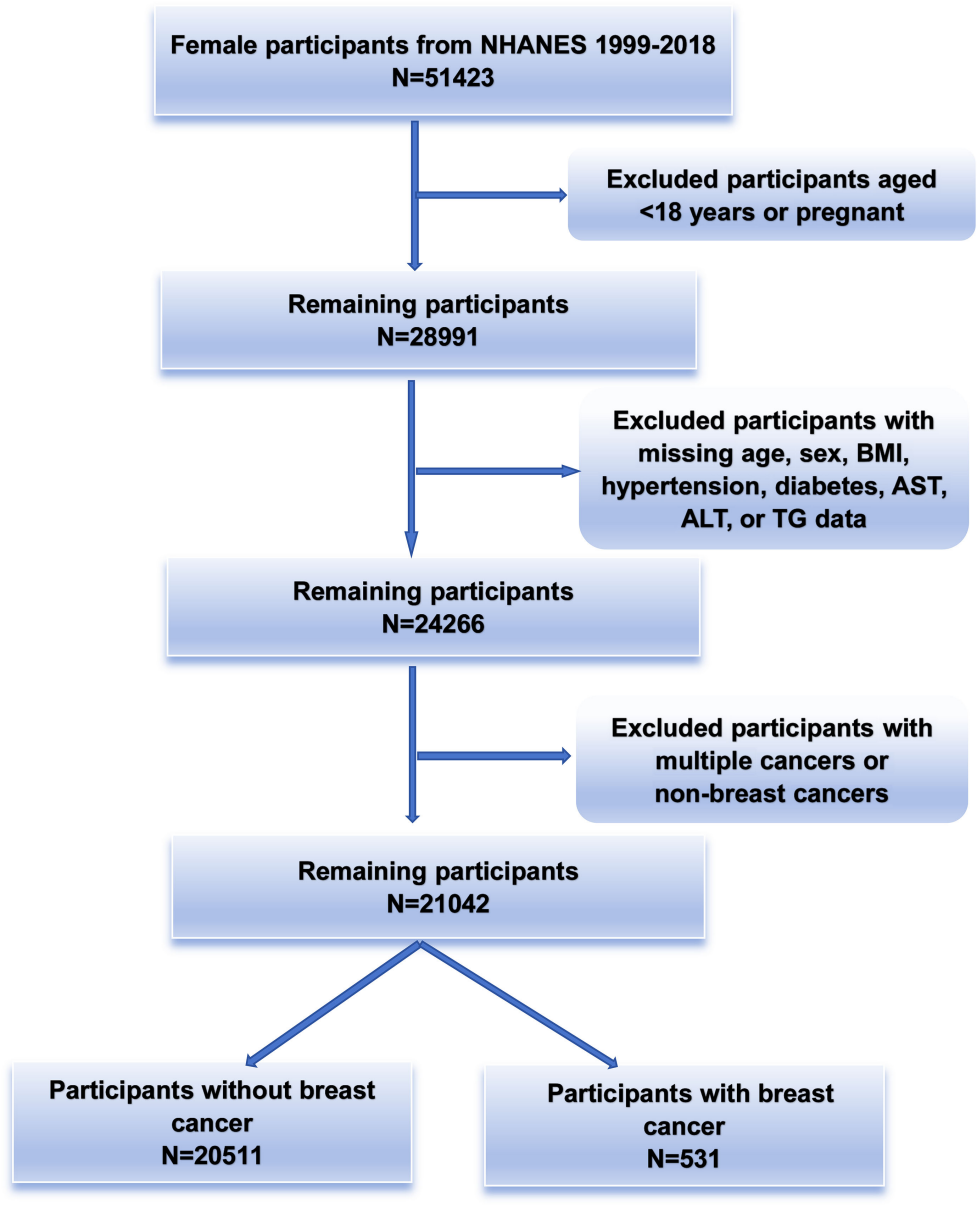
Flowchart of participant selection for the NHANES 1999–2018 analysis.

For the present analysis, we limited the group to adult women with ages 18 years or older who had complete data on metabolic biomarkers (including triglycerides (TG), alanine aminotransferase (ALT), and aspartate aminotransferase (AST)), anthropometric measurements (body mass index (BMI)), and variables required for the calculation of FSI as well as for covariate adjustment. Participants were excluded if they were younger than 18 years, were pregnant during the examination period, reported a history of malignancies other than breast cancer, lacked information on breast cancer status, or had missing data for key FSI components (sex, age, BMI, diabetes, hypertension, TG, ALT, or AST). After applying these criteria, the final analytical sample comprised 21,042 women (**Figure 1**).

### Study variables

#### Definition of breast cancer

Breast cancer status was ascertained using standardized NHANES questionnaire data. Participants were classified according to their responses to two NHANES questionnaire items (1): whether they had ever been told by a doctor or other health professional that they had cancer or a malignancy, and, if yes (2), what kind of cancer it was. Women who self-reported a physician diagnosis of breast cancer were classified as having breast cancer. Women who reported no history of cancer were classified as non-cases. Women with a history of malignancies other than breast cancer were excluded during participant selection. Relevant questionnaire items and coding procedures are publicly available in the NHANES documentation.

#### Definition of FSI

The FSI was calculated using the original published formula (12):

FSI = -7.981 + 0.011 × age (years) - 0.146 × sex (female = 1, male = 0) + 0.173 × BMI (kg/m2) + 0.007 × triglycerides (mg/dL) + 0.593 × hypertension (yes = 1, no = 0) + 0.789 × diabetes (yes = 1, no = 0) + 1.1 × ALT/AST ratio ≥ 1.33 (yes = 1, no = 0).

Triglycerides were converted from mmol/L to mg/dL using the factor of 88.57. Age and sex were obtained from the demographic component; BMI and blood pressure were obtained from the examination component; and serum TG, ALT, and AST were obtained from laboratory data. Diabetes was defined as self-reported physician diagnosis with current anti-diabetic treatment, fasting plasma glucose ≥ 7.0 mmol/L, HbA1c ≥ 6.5%, or random plasma glucose ≥ 11.1 mmol/L. Hypertension was defined as mean systolic blood pressure ≥ 140 mmHg, mean diastolic blood pressure ≥ 90 mmHg, current antihypertensive treatment, or self-reported physician diagnosis of hypertension.

#### Definition of covariates

Covariates were selected *a priori* based on epidemiological relevance and data availability and included race/ethnicity, educational attainment, family income-to-poverty ratio (PIR), marital status, smoking status, and alcohol consumption. Race/ethnicity was categorized as Mexican American, other Hispanic, non-Hispanic White, non-Hispanic Black, and other race. Educational attainment was categorized as less than high school, high school or equivalent, and college or above. Marital status was categorized as married/living with partner, widowed/divorced/separated, never married, or other. Smoking status was categorized as no (<100 cigarettes in a lifetime), yes (≥100 cigarettes in a lifetime), or other. Alcohol consumption was categorized as no (<12 drinks in a lifetime), yes (≥12 drinks in a lifetime), or other. These variables were included in the multivariable models as potential confounders.

### Exploratory hospital-based cohort

To provide exploratory supportive evidence for the NHANES findings, we analyzed a hospital-based cohort. This cohort included consecutive adult women who underwent breast ultrasound and/or mammography evaluation at Guangxi Medical University Cancer Hospital between January and November 2025. Eligibility criteria were designed to broadly parallel the NHANES analysis. We excluded women who were younger than 18 years, were pregnant, had a previous or concurrent non-breast malignancy, lacked definitive breast lesion classification, or had missing information required for FSI calculation. Breast cancer cases were women with primary breast cancer confirmed histopathologically. Controls were women with benign breast nodules and no prior history of malignancy. A total of 160 participants were included in this exploratory cohort.

Within this cohort, FSI was calculated using the same formula as in the NHANES analysis. Clinical and demographic variables were abstracted from routinely collected records at the index visit. For breast cancer cases, clinical and laboratory variables used for FSI calculation were obtained at the index visit before anti-tumor treatment. Because some NHANES covariates, such as PIR, were not routinely available in the hospital setting, only locally available variables were extracted, including age, ethnicity, educational attainment, marital status, smoking history, and alcohol consumption. Ethnicity was retained as a locally available sociocultural covariate rather than as a direct analogue of NHANES race/ethnicity.

This study was approved by the Institutional Review Board of Guangxi Medical University Cancer Hospital (KY2026424).

### Statistical analysis

Analyses were executed in R (version 4.4.1) and EmpowerStats (version 4.2). A two-sided P value < 0.05 was considered statistically significant. For the NHANES analysis, examination weights were incorporated to account for the multi-cycle survey weighting structure. When combining survey cycles from 1999 to 2018, the 2-year examination weights (WTMEC2YR) were rescaled by dividing by the number of combined 2-year cycles to generate appropriate multi-cycle weights. Baseline characteristics were presented as survey-weighted means ± standard deviations (SDs) for continuous variables and weighted proportions for categorical variables. Associations between FSI and prevalent breast cancer were evaluated using multivariable logistic regression models, with results reported as odds ratios (ORs) and 95% confidence intervals (CIs). FSI was analyzed both as a continuous variable and by quartiles. Three sequential models were constructed: Model 1, unadjusted; Model 2, adjusted for race/ethnicity and educational attainment; and Model 3, additionally adjusted for PIR, marital status, smoking status, and alcohol consumption. Tests for linear trend across FSI quartiles were conducted by entering the median value of each quartile as a continuous variable. Because age is included in the FSI formula, we performed an additional sensitivity analysis by further adjusting Model 3 for continuous age. The results were presented in **Supplementary Table 2**.

To address whether the association between FSI and prevalent breast cancer might be driven by individual components of the FSI, we performed additional component-level analyses in the NHANES analytic sample. FSI and its individual components, including age, BMI, triglycerides, diabetes, hypertension, and ALT/AST ratio ≥1.33, were separately examined in weighted logistic regression models adjusted for the same covariates as Model 3. Continuous variables were standardized to allow comparison of effect estimates per 1-SD increment. We further compared the discriminatory performance of the base model, the base model plus FSI, and the base model plus individual FSI components by calculating the area under the receiver operating characteristic curve (AUC). These analyses were used to clarify component contributions, not to infer an FSI effect independent of its constituent variables.

Potential non-linear associations were assessed using restricted cubic spline (RCS) analysis and generalized additive models (GAMs). For the main NHANES RCS and threshold analyses, 95 participants with extreme FSI values (FSI > 5) were excluded to reduce the influence of sparse extreme observations on graphical display and non-linear model estimation; the corresponding restricted analytic sample included 20,947 participants. When non-linearity was suggested, a two-piecewise logistic regression model was applied to identify a potential inflection point, and model fit was compared with that of a linear model using a likelihood ratio test. Predefined subgroup analyses and interaction tests were performed to evaluate possible effect modification. Estimates from sparse residual categories were interpreted cautiously.

For the exploratory hospital-based cohort, logistic regression models were constructed using an analogous modeling strategy, with adjustment limited to covariates available in the local dataset. For exploratory comparability, quartile analyses in the hospital-based cohort used the same FSI cut points as those defined in the NHANES cohort. Because these cut points were derived from the NHANES distribution rather than from the hospital-based cohort itself, the categorical results in the exploratory cohort were interpreted cautiously.

## Results

### Baseline characteristics of participants

Among the 21,042 women included in the NHANES analysis, 531 (2.5%) reported a history of breast cancer. As shown in **Table 1**, women with prevalent breast cancer were older than those without breast cancer (64.93 ± 12.22 vs. 46.68 ± 16.63 years, P < 0.0001). They were also more likely to be non-Hispanic White and to have a higher household income. In addition, women with breast cancer had higher FSI values than those without breast cancer (−0.84 ± 1.72 vs. −1.31 ± 1.88, P < 0.0001), as well as higher prevalences of hypertension and diabetes. Triglyceride levels also differed significantly between the two groups, although the absolute difference was small. No significant between-group differences were observed for educational attainment, smoking status, BMI, AST, or ALT.

**Table 1:** Baseline characteristics of female participants in NHANES 1999–2018 according to prevalent breast cancer status.

Characteristics	Total	Breast cancer status		*P* value
	(N = 21042)	No(N = 20511)	Yes(N = 531)	
Age (years)	47.14 ± 16.78	46.68 ± 16.63	64.93 ± 12.22	<0.0001
Race/ethnicity (%)				<0.0001
Mexican American	7.77	7.89	3.26	
Other Hispanic	5.96	6.04	2.76	
Non-Hispanic White	67.31	66.90	82.95	
Non-Hispanic Black	11.92	12.04	7.11	
Other	7.05	7.13	3.92	
Education (%)				0.7262
Less than high school	16.53	16.57	15.10	
High school or equivalent	23.36	23.34	24.24	
College or above	60.03	60.01	60.66	
Other	0.08	0.08	-	
Marital status (%)				<0.0001
Married/living with partner	59.74	59.80	57.44	
Never married	16.29	16.59	4.85	
Widowed/Divorced/Separated	22.95	22.58	37.00	
Others	1.02	1.03	0.71	
PIR (%)				<0.0001
Low	15.46	15.60	10.18	
Moderate	37.12	37.21	33.48	
High	47.42	47.19	56.34	
Drinking status (%)				<0.0001
No	14.42	14.31	18.66	
Yes	23.98	23.77	32.06	
Other	61.61	61.93	49.28	
Smoking status (%)				0.3078
No	61.14	61.22	57.98	
Yes	38.80	38.72	41.98	
Other	0.06	0.06	0.04	
Hypertension (%)				<0.0001
No	65.99	66.56	44.17	
Yes	34.01	33.44	55.83	
Diabetes (%)				<0.0001
No	85.42	85.74	73.10	
Yes	14.58	14.26	26.90	
BMI(kg/m²)	28.90 ± 7.39	28.90 ± 7.41	28.97± 6.50	0.4532
TG(mmol/L)	1.47 ± 1.07	1.48 ± 1.08	1.46± 1.00	0.0014
AST(U/L)	23.11 ± 13.23	23.10 ± 13.34	23.55 ± 7.91	0.4369
ALT(U/L)	21.23 ± 20.67	21.24 ± 20.89	20.66 ± 8.84	0.5217
FSI(score)	-1.30 ± 1.87	-1.31 ± 1.88	-0.84 ± 1.72	<0.0001

Values are presented as survey-weighted mean ± SD for continuous variables and weighted percentages for categorical variables. FSI, Framingham steatosis index; PIR, family income-to-poverty ratio; BMI, body mass index; TG, triglycerides; AST, aspartate aminotransferase; ALT, alanine aminotransferase.

In the exploratory hospital-based cohort, 109 of 160 participants (68.1%) had breast cancer. As summarized in **Supplementary Table 4**, women with breast cancer were older (43.81 ± 7.51 vs. 37.00 ± 9.87 years, P < 0.001), had higher BMI, and had higher FSI values (−2.78 ± 1.05 vs. −3.29 ± 0.85, P = 0.001) than controls. Educational attainment (P < 0.001) and marital status (P = 0.002) also differed significantly between groups. In contrast, ethnicity, alcohol consumption, smoking status, hypertension, diabetes, TG, AST, and ALT were not significantly different between the two groups.

### Association between the FSI and prevalent breast cancer

Before fitting the regression models, we assessed multicollinearity among the covariates. All variables included in the models had variance inflation factors <5, indicating no evidence of substantial collinearity (**Supplementary Table 1**).

Because breast cancer status in NHANES reflected self-reported prevalent disease rather than incident breast cancer, these estimates should be interpreted as associations with breast cancer history rather than evidence that higher FSI preceded breast cancer development. As shown in **Table 2**, higher FSI values were associated with higher odds of prevalent breast cancer in the NHANES cohort. When FSI was analyzed as a continuous variable, each 1-unit increase in FSI was associated with a 12% increase in the odds of prevalent breast cancer in the unadjusted model (OR 1.12, 95% CI 1.07–1.16, P < 0.0001). This association remained significant after adjustment for race/ethnicity and educational attainment (OR 1.13, 95% CI 1.08–1.18, P < 0.0001) and after further adjustment for PIR, marital status, smoking status, and alcohol consumption (OR 1.10, 95% CI 1.05–1.16, P < 0.0001). When FSI was categorized into quartiles, participants in higher FSI quartiles generally had higher odds of prevalent breast cancer than those in the lowest quartile, with a significant trend across quartiles. In the fully adjusted model, compared with the lowest quartile, the ORs for prevalent breast cancer were 1.35 (95% CI 1.01–1.79) for Q2, 1.80 (95% CI 1.37–2.36) for Q3, and 1.72 (95% CI 1.30–2.27) for Q4. These findings suggested a positive association in the primary model.

**Table 2:** Association between the FSI and prevalent breast cancer in female participants from NHANES 1999–2018.

Exposure	OR (95% CI), p value		
	Model 1*	Model 2*	Model 3*
FSI, continuous	1.12 (1.07, 1.16), <0.0001	1.13 (1.08, 1.18), <0.0001	1.10 (1.05, 1.16), <0.0001
FSI quartiles			
Q1 (<-2.52)	reference	reference	reference
Q2(-2.52 - -1.29)	1.46 (1.10, 1.93), 0.0081	1.55 (1.17, 2.06),0.0021	1.35 (1.01, 1.79),0.0403
Q3(-1.29- 0.08)	2.00 (1.53, 2.60), <0.0001	2.20 (1.68, 2.87), <0.0001	1.80 (1.37, 2.36), <0.0001
Q4 (≥0.08)	1.86 (1.42, 2.43), <0.0001	2.07 (1.57, 2.71), <0.0001	1.72 (1.30, 2.27), 0.0001
P for trend	<0.0001	<0.0001	<0.0001

Model 1 was unadjusted. Model 2 was adjusted for race/ethnicity and educational attainment. Model 3 was additionally adjusted for PIR, marital status, smoking status, and alcohol consumption.

Because age is directly included in the FSI formula, we further performed an age-adjusted sensitivity analysis. After additional adjustment for continuous age on the basis of Model 3, the association between FSI and prevalent breast cancer was substantially attenuated and was no longer statistically significant. For continuous FSI, the OR was 1.02 (95% CI: 0.96–1.07, P = 0.5263). In the quartile analysis, no monotonic dose-response trend was observed after age adjustment (P for trend = 0.8012; **Supplementary Table 2**). These findings suggest that the association observed in the primary model was substantially influenced by the age component of FSI.

To further evaluate whether the association was driven by individual FSI components, we performed component-level analyses (**Supplementary Table 3**). Age showed the strongest association and discriminatory performance among the FSI components. Hypertension, diabetes, and triglycerides were also associated with prevalent breast cancer, whereas BMI and ALT/AST ratio ≥1.33 were not. Adding FSI to the base model produced only a modest increase in AUC, while adding age produced the largest improvement. These findings suggest that the FSI–breast cancer association was mainly influenced by age and selected metabolic components rather than by BMI alone or an isolated hepatic steatosis effect.

In the exploratory hospital-based cohort, continuous FSI was positively associated with breast cancer case status in the fully adjusted model (OR 1.65, 95% CI 1.07–2.66, P = 0.03; **Supplementary Table 5**). However, the quartile-based trend was no longer significant after full adjustment (P for trend = 0.106). Given the small sample size, NHANES-derived quartile cut points, and reduced covariate availability, these findings provide only partial supportive evidence and do not reproduce the NHANES quartile-based dose-response pattern.

### Non-linear relationship between FSI and prevalent breast cancer

RCS analysis was chosen as the primary approach in the NHANES cohort to allow formal testing of non-linearity and threshold effects, whereas GAM was applied in the exploratory hospital-based cohort due to the smaller sample size and narrower exposure distribution.

**Figure 2:**
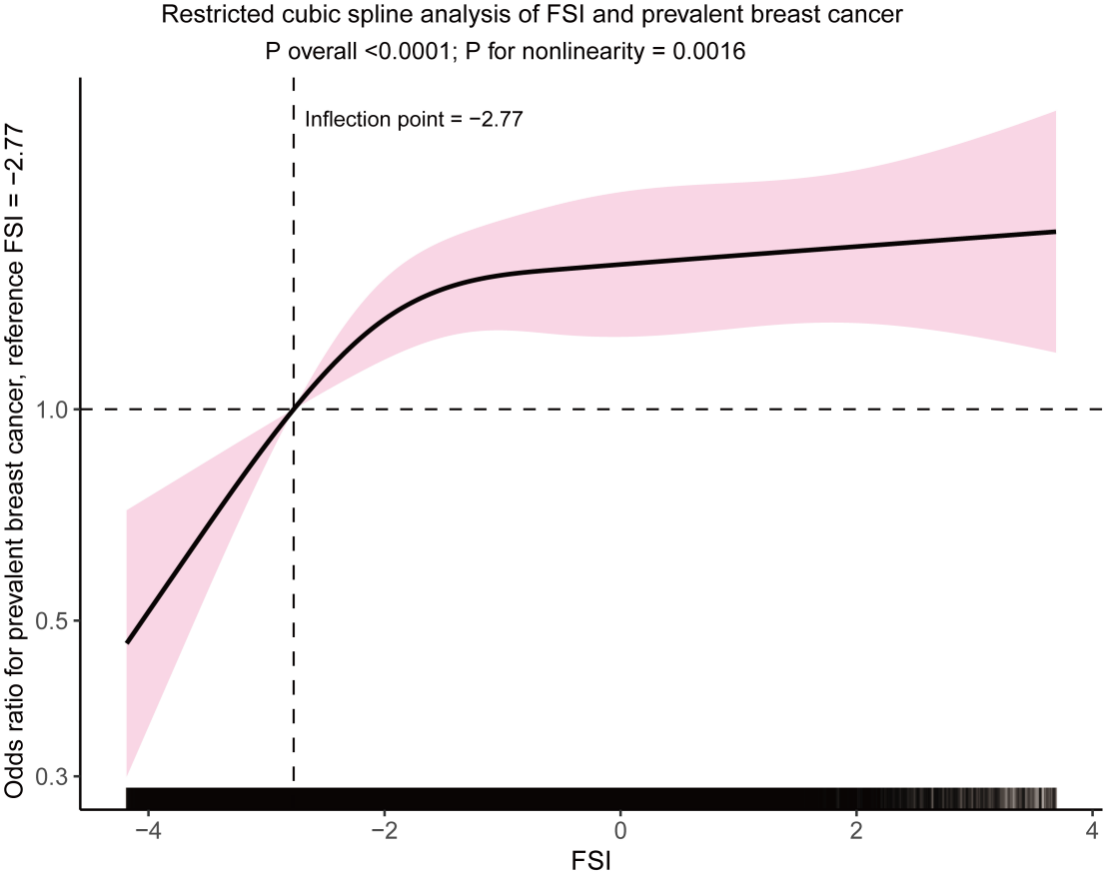
Restricted cubic spline analysis of the association between the Framingham Steatosis Index and prevalent breast cancer in NHANES 1999–2018. The analysis was conducted after excluding 95 participants with extreme FSI values (FSI > 5), leaving 20,947 participants. The curve was fitted using the same covariate adjustment scheme as the threshold analysis, with FSI = −2.77 set as the reference value. The vertical dashed line indicates the estimated inflection point. The solid line represents the adjusted odds ratio, and the shaded area indicates the 95% confidence interval. The spline curve displays pointwise ORs relative to FSI = −2.77 across the continuous FSI distribution; these estimates should be interpreted separately from the segment-specific ORs reported in **Table 3**.

In the NHANES cohort, after adjustment for the same covariates as in the fully adjusted logistic regression model, the initial full-sample analysis (N = 21,042) suggested a non-linear association between FSI and prevalent breast cancer. The curve indicated that the odds of prevalent breast cancer increased more steeply at lower FSI levels and more gradually at higher levels. However, estimates at the upper end of the FSI distribution were less precise, as reflected by wider confidence intervals, likely because relatively few participants had very high FSI values. Therefore, to reduce the influence of sparse extreme FSI values on the graphical display and non-linear model estimation, 95 participants with FSI > 5 were excluded, leaving 20,947 participants for the main RCS analysis shown in **Figure 2**. In this refitted spline model, a similar non-linear pattern was observed, and the tests remained statistically significant (P overall < 0.0001; P for non-linearity = 0.0016).

**Figure 3:**
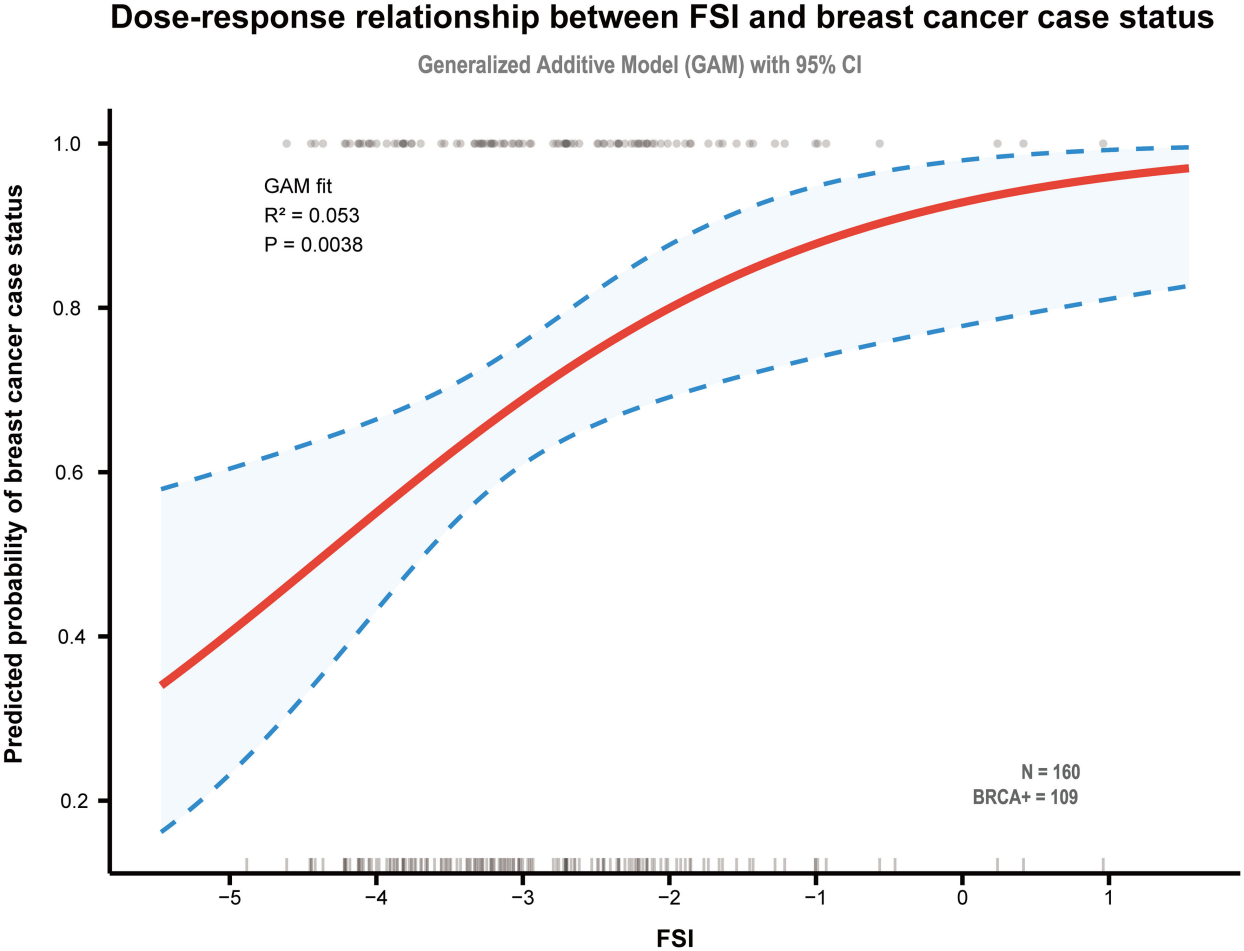
Generalized additive model of the association between the Framingham Steatosis Index and breast cancer case status in the exploratory hospital-based cohort. The solid line represents the fitted curve, and the dashed lines indicate the 95% confidence interval.

In the exploratory hospital-based cohort, GAM was used to further assess the exposure-response pattern. The fitted curve suggested a positive but approximately linear association between FSI and breast cancer case status (P = 0.0038), without clear evidence of an inflection point (**Figure 3**). This likely reflects the narrower exposure range and limited statistical power of the exploratory cohort, within which the association appeared to be adequately captured by a linear specification.

For completeness, GAM-based curves for the NHANES cohort are provided in **Supplementary Figure S1** and showed patterns generally consistent with the main RCS analysis.

### Threshold effect analysis in NHANES and the exploratory hospital-based cohort

To further characterize the non-linear association observed in NHANES, we fitted a two-piecewise logistic regression model. This analysis identified a potential inflection point at FSI = -2.77 (**Table 3**). Below this value, each 1-unit increase in FSI was associated with substantially higher odds of prevalent breast cancer (OR 3.11, 95% CI 1.82–5.30, P < 0.0001). Above the inflection point, the association remained positive but was markedly attenuated (OR 1.06, 95% CI 1.00–1.12, P = 0.0352). Comparison of model fit using the likelihood ratio test showed that the two-piecewise model fit the data better than the standard linear model (P < 0.001), suggesting a possible threshold-like pattern in the NHANES cohort. However, this threshold analysis was considered exploratory and should not be interpreted as establishing a biologically defined cutoff.

**Table 3:** Exploratory threshold effect analysis of the association between the Framingham Steatosis Index and prevalent breast cancer in NHANES 1999–2018 after exclusion of extreme FSI values.

Outcome	OR (95% CI), p value
Fitting by standard logistic model	1.12 (1.07, 1.18), P <0.0001
Fitting by two-piecewise logistic regression model	
Inflection point	-2.77
< -2.77	3.11 (1.82, 5.30) <0.0001
> -2.77	1.06 (1.00, 1.12) 0.0352
Likelihood ratio test P value	<0.001

This threshold analysis was conducted in the restricted analytic sample after exclusion of 95 participants with extreme FSI values (FSI > 5), leaving 20,947 participants. The model was adjusted for race/ethnicity, educational attainment, smoking status, marital status, alcohol consumption, and family income-to-poverty ratio. The standard logistic estimate in this table may differ slightly from the continuous FSI estimate in **Table 2** because **Table 3** was based on the restricted analytic sample. The ORs from the two-piecewise logistic regression represent segment-specific OR estimates per 1-unit increase in FSI within each interval and should not be interpreted as numerically equivalent to the pointwise ORs displayed by the restricted cubic spline curve.

In the exploratory hospital-based cohort, the two-piecewise model did not fit significantly better than the linear model (likelihood ratio test P = 0.2519; **Supplementary Table 6**), consistent with the approximately linear GAM curve. Thus, the NHANES threshold-like pattern should be regarded as hypothesis-generating and requires confirmation in larger independent datasets.

### Subgroup analysis

We conducted subgroup analyses to evaluate whether the association between FSI and prevalent breast cancer varied across demographic and lifestyle strata (**Table 4**). Overall, the association between higher FSI and higher odds of prevalent breast cancer was directionally consistent across most subgroups. Formal interaction tests did not identify statistically significant effect modification by race/ethnicity, educational attainment, marital status, PIR, smoking status, or alcohol consumption (all P for interaction > 0.05). Although the estimated association appeared numerically stronger among women with a college education or higher, there was no statistically significant evidence of heterogeneity across educational strata.

**Table 4:** Subgroup analyses of the association between the Framingham Steatosis Index and prevalent breast cancer in NHANES 1999–2018.

Subgroup	OR, (95% CI)	P value	P interaction
Race/ethnicity			0.5117
Mexican American	1.21 (1.06, 1.38)	0.0055	
Other Hispanic	1.12 (0.94, 1.34)	0.2011	
Non-Hispanic White	1.07 (1.01, 1.14)	0.0204	
Non-Hispanic Black	1.15 (1.02, 1.28)	0.0171	
Other	1.13 (0.94, 1.36)	0.1801	
Education			0.3303
Less than high school	1.07 (0.96, 1.18)	0.2106	
High school or equivalent	1.04 (0.95, 1.15)	0.3883	
College or above	1.15 (1.08, 1.22)	<0.0001	
Other	–	–	
Marital status			0.2205
Married/living with partner	1.12 (1.06, 1.19)	0.0002	
Never married	1.23 (1.06, 1.44)	0.0080	
Widowed/Divorced/ Separated	1.06 (0.98, 1.14)	0.1379	
PIR			0.8368
Low	1.13 (1.02, 1.24)	0.0149	
Moderate	1.09 (1.01, 1.17)	0.0224	
High	1.11 (1.03, 1.19)	0.0051	
Drinking status			0.5263
Yes	1.06 (0.98, 1.15)	0.1594	
No	1.11 (1.00, 1.23)	0.0508	
Other	1.13 (1.06, 1.20)	0.0002	
Smoking status			0.5170
Yes	1.07 (1.00, 1.15)	0.0464	
No	1.13 (1.06, 1.20)	<0.0001	
Other	1.13 (0.27, 4.83)	0.8654	

Odds ratios were estimated per 1-unit increase in FSI. Subgroup-specific estimates for sparse residual categories were not emphasized because of unstable model estimates.

Given the limited sample size and reduced statistical power of the exploratory hospital-based cohort, subgroup analyses were not performed in that cohort.

## Discussion

In this study, we evaluated the association between the Framingham Steatosis Index (FSI) and prevalent breast cancer in women using a large NHANES dataset and an exploratory hospital-based cohort. In the primary NHANES models, higher FSI values were associated with higher odds of prevalent breast cancer after adjustment for demographic, socioeconomic, and lifestyle factors. However, this association was substantially attenuated and became non-significant after additional adjustment for age, indicating that the observed association was strongly influenced by the age component of FSI. Non-linear modeling further suggested that this association was not uniform across the full FSI distribution, with a steeper increase at lower FSI levels and a more gradual increase beyond the identified inflection point. Although the spline pattern remained similar after excluding extreme values, these findings should be interpreted cautiously given the cross-sectional design, the composite nature of FSI, and the attenuation after age adjustment.

In the exploratory hospital-based cohort, continuous FSI was positively associated with breast cancer case status, but the quartile-based dose-response and threshold-like pattern observed in NHANES were not reproduced. Given its small sample size, selected hospital-based population, narrower FSI range, unbalanced case-control distribution, and fewer available covariates, this cohort should be regarded as exploratory and supportive rather than confirmatory. Its main value is that the continuous association showed a similar direction in a separate clinical setting, but it does not constitute external validation of the NHANES dose-response pattern.

Although the present study was not designed to establish specific biological mechanisms, the observed association is biologically plausible within the broader framework of metabolic dysfunction and hepatic steatosis-related phenotypes. FSI was originally developed as a practical non-invasive surrogate marker of hepatic steatosis and integrates multiple interrelated components, including age, adiposity, triglycerides, diabetes, hypertension, and the ALT/AST ratio (12, 13). Therefore, rather than representing the effect of a single metabolic abnormality, FSI should be interpreted in the present cancer epidemiology context as a composite marker of age-related metabolic-hepatic burden. Component-level analyses further supported this interpretation: age was the dominant contributor, while hypertension, diabetes, and triglycerides also contributed; in contrast, BMI and ALT/AST ratio ≥1.33 were not significantly associated with prevalent breast cancer. Thus, the observed association cannot be attributed specifically to hepatic steatosis itself or to BMI alone.

Among the metabolic domains represented by FSI, adiposity is particularly relevant to breast cancer biology. Previous studies have shown that higher BMI and obesity are related to breast cancer risk, especially around and after menopause, although the magnitude and direction of the association may vary according to hormonal context (14, 15). Mechanistically, excess adiposity may contribute through increased estrogen biosynthesis, insulin resistance, IGF-1 signaling, adipokine dysregulation, oxidative stress, and chronic low-grade inflammation (14, 16–18). Diabetes may further amplify this pro-tumorigenic environment through hyperglycemia, compensatory hyperinsulinemia, insulin/IGF-1 signaling, oxidative stress, and inflammatory activation (19). Triglyceride-related metabolic indices have also been linked to breast cancer occurrence or outcomes in previous population-based studies, likely reflecting insulin resistance, altered lipid availability, and broader metabolic dysregulation (20–22). Hypertension, another component of FSI, may represent part of a broader vascular, inflammatory, endocrine, and cardiometabolic phenotype rather than an isolated breast cancer-specific pathway (23).

Hepatic steatosis-related phenotypes may provide an additional link between systemic metabolic dysfunction and extrahepatic malignancies. Prior studies have reported associations between fatty liver-related indices, NAFLD or MASLD, and extrahepatic cancers, including breast cancer (7–11). However, because hepatic steatosis commonly coexists with obesity, insulin resistance, dyslipidemia, diabetes, and hypertension, the present data cannot distinguish an isolated hepatic steatosis effect from the broader metabolic context captured by FSI. Accordingly, FSI may be useful as a pragmatic summary indicator for characterizing metabolic-hepatic burden in epidemiological studies, but it should not be treated as a direct diagnostic measure of fatty liver or as an exposure independent of its constituent variables.

The non-linear and threshold analyses should also be interpreted cautiously. In NHANES, the RCS analysis suggested a non-linear association, and the two-piecewise model identified a potential inflection point at FSI = −2.77. One possible explanation is that, at relatively low FSI levels, incremental worsening in metabolic-hepatic burden may correspond to a sharper increase in the odds of prevalent breast cancer, whereas at higher FSI levels the marginal increase becomes smaller. However, this pattern may also be influenced by the distribution of FSI values, sparse observations at the upper exposure range, and model specification. The absence of a similar threshold-like pattern in the exploratory hospital-based cohort further supports treating the threshold result as hypothesis-generating rather than as a biologically established cutoff.

Several strengths of this study merit consideration. First, the NHANES analysis was based on a large population-based sample collected using standardized procedures, which supports the descriptive reliability of the primary findings. Second, FSI was evaluated as both a continuous and categorical exposure, and flexible non-linear modeling was used to characterize the exposure-response pattern beyond a simple linear assumption. Third, the age-adjusted sensitivity analysis and component-level/AUC analyses directly addressed the composite nature of FSI and helped clarify the relative contribution of age and selected metabolic components. Fourth, the exploratory hospital-based cohort provided additional clinical context using pre-treatment measurements in breast cancer cases, although it was not designed or powered to serve as confirmatory external validation.

This study has several limitations. First, because NHANES is cross-sectional, temporal relationships cannot be established and reverse causation cannot be excluded. Second, breast cancer status in NHANES was based on self-reported physician diagnosis and therefore may be subject to misclassification. Third, the outcome definitions were not fully identical across datasets: NHANES captured self-reported prevalent breast cancer, whereas the exploratory hospital-based cohort evaluated contemporaneous histopathologically confirmed breast cancer case status. Fourth, prevalence-based analyses may also be influenced by survivorship bias, selection bias related to the exclusion criteria and complete-case analysis, and metabolic alterations related to prior cancer diagnosis or treatment. Fifth, residual confounding remains possible because reproductive, hormonal, familial/genetic, lifestyle, treatment-related, and molecular subtype information, including ER, PR, HER2, and Ki-67 status, was unavailable, incomplete, or not consistently harmonized across datasets. In addition, FSI is a composite index that incorporates age and several metabolic variables. Although component-level and discrimination analyses were conducted, the present study cannot disentangle the independent contribution of hepatic steatosis from that of age, hypertension, diabetes, triglycerides, BMI, and liver enzyme-related components. Therefore, FSI should be interpreted as a composite metabolic-hepatic marker rather than as a fatty liver-specific exposure. Finally, the exploratory hospital-based cohort was relatively small and had fewer available covariates, which limited statistical precision and reduced our ability to fully examine non-linearity and effect modification. Therefore, the hospital-based findings should be interpreted as exploratory and supportive rather than confirmatory.

## Conclusion

Higher FSI was associated with prevalent breast cancer status in the primary NHANES analysis, but this association was substantially attenuated after additional adjustment for age. Component-level analyses indicated that the association was mainly influenced by age and selected metabolic components. Therefore, FSI should be interpreted as a composite marker of age-related metabolic-hepatic burden rather than as evidence of an independent hepatic steatosis effect or an age-independent, causal, or diagnostic biomarker. Prospective studies with incident breast cancer outcomes, treatment information, reproductive and hormonal variables, and molecular subtype data are needed to clarify temporality and component-specific contributions.

## Data Availability

The NHANES datasets analyzed in this study are publicly available. The de-identified data from the exploratory hospital-based cohort are available from the corresponding author upon reasonable request, subject to institutional and ethical restrictions.
