# Supplementary material for: Higher Framingham steatosis index is associated with prevalent breast cancer in women: cross-sectional evidence from NHANES 1999–2018 and an exploratory hospital-based dataset
Source: Front Oncol. 2026 Jul 8;16:1849341. doi: 10.3389/fonc.2026.1849341 (PMC13388184; doi:10.3389/fonc.2026.1849341)
Supplement: Supplementary file 1 [file DataSheet1.pdf]

Supplementary Tables and Figures

Table legends

Supplementary Table S1 Multicollinearity diagnostics for variables included in the NHANES regression models

variables	VIF
FSI	1.05
Marital status	1.06
Education	1.27
Poverty-to-income ratio	1.22
Race	1.09
Drinking	1.13
Smoking	1.10

Note: Variance inflation factor (VIF) values were used to assess multicollinearity. Variables with VIF > 5 were considered to indicate substantial collinearity.

Supplementary Table S2 Sensitivity analysis with additional adjustment for continuous age.

Exposure	OR (95% CI), P value
	Model 4*
FSI(Continuous)	1.02 (0.96, 1.07), 0.5263
FSI quartiles	
Q1 (<-2.52)	reference
Q2(-2.52 - -1.29)	0.71 (0.53, 0.96), 0.0239
Q3(-1.29- 0.08)	0.81 (0.61, 1.08), 0.1492
Q4 (≥0.08)	0.84 (0.63, 1.12), 0.2444
P for trend	0.8012

Note: Model 4 was additionally adjusted for Model 3 plus age.

Supplementary Table S3. Component-level associations and discriminatory performance of FSI and its individual components for prevalent breast cancer in NHANES 1999–2018.

Variable/model	OR (95% CI)	P value	AUC	ΔAUC vs base model
Base model	—	—	0.600	0.000
FSI, per 1-SD increase	1.23 (1.14, 1.34)	<0.001	0.619	0.019

Variable/model	OR (95% CI)	P value	AUC	ΔAUC vs base model
Age, per 1-SD increase	3.64 (3.27, 4.05)	<0.001	0.795	0.195
BMI, per 1-SD increase	0.96 (0.88, 1.05)	0.390	0.600	0.000
Triglycerides, per 1-SD increase	1.10 (1.03, 1.17)	0.004	0.607	0.007
Diabetes, yes vs no	2.11 (1.73, 2.58)	<0.001	0.628	0.028
Hypertension, yes vs no	2.38 (2.00, 2.84)	<0.001	0.657	0.057
ALT/AST ratio ≥1.33, yes vs no	0.82 (0.55, 1.20)	0.308	0.601	0.001
Base model + all FSI components	—	—	0.796	0.196

Note: Models were adjusted for race/ethnicity, educational attainment, PIR, marital status, smoking status, and alcohol consumption. Continuous variables were modeled per 1-SD increment. AUC values were calculated for the base model and models additionally incorporating FSI or individual FSI components. These analyses were conducted to compare the contribution of FSI components and were not intended to establish an independent effect of FSI beyond its constituent variables.

Abbreviations: AUC, area under the receiver operating characteristic curve; BMI, body mass index; FSI, Framingham Steatosis Index; PIR, family income-to-poverty ratio; SD, standard deviation; TG, triglycerides.

Supplementary Table S4. Baseline characteristics of the exploratory hospital-based cohort according to breast cancer status.

Characteristics	Total (N=160)	Breast cancer status<br>No(N=51)	Yes(N=109)	*P* value
Age (years)	41.64 ± 8.89	37.00 ± 9.87	43.81 ± 7.51	<0.001
Ethnicity (%)				0.263
Han	63.13	56.86	66.06	
Zhuang	32.50	35.29	31.19	
Other	4.38	7.84	2.75	
Education (%)				<0.001
High School or Below	31.88	19.61	37.61	
College and Above	33.13	23.53	37.61	
Other	35.00	56.86	24.77	
Marital status (%)				0.002
married	85.00	76.47	88.99	

Characteristics	Total (N=160)	Breast cancer status		
		No(N=51)	Yes(N=109)	*P* value
Never married	11.25	23.53	5.50	
Others	3.75	0.00	5.50	
Drinking status (%)				0.078
No	81.88	90.20	77.98	
Yes	18.13	9.80	22.02	
Smoking status (%)				>0.999
No	99.38	100.00	99.08	
Yes	0.63	0.00	0.92	
Hypertension (%)				0.051
No	85.63	94.12	81.65	
Yes	14.38	5.88	18.36	
Diabetes (%)				0.178
No	96.25	100.00	94.50	
Yes	3.75	0.00	5.50	
BMI(kg/m²)	22.69 ± 3.37	21.74 ± 2.90	23.14 ± 3.49	0.009
TG(mg/dL)	88.95 ± 52.88	83.97 ± 46.33	91.28 ± 55.73	0.386
AST(U/L)	20.99 ± 7.27	19.76 ± 5.76	21.56 ± 7.84	0.106
ALT(U/L)	16.63 ± 10.20	15.51 ±9.07	17.16 ± 10.69	0.315
FSI(score)	-2.94 ± 1.02	-3.29 ± 0.85	-2.78 ± 1.05	0.001

Note: Values are presented as mean ± SD for continuous variables and percentages for categorical variables.

Supplementary Table S5. Association between the Framingham Steatosis Index and breast cancer case status in the exploratory hospital-based cohort.

Exposure	OR (95% CI), P value		
	Model 1*	Model 2*	Model 3*
FSI(Continuous)	1.80(1.23, 2.75), 0.004	1.95(1.30, 3.07), 0.002	1.65(1.07, 2.66), 0.03
FSI quartiles			
Q1 (<-2.52)	Reference	Reference	Reference
Q2(-2.52 - -1.29)	0.66 (0.27, 1.61), 0.37	0.89 (0.34, 2.33), 0.80	0.54 (0.17, 1.58), 0.27
Q3(-1.29- 0.08)	1.80 (0.70, 4.81), 0.23	1.86 (0.68, 5.30), 0.23	1.06 (0.34, 3.31), 0.92
Q4 (≥0.08)	2.83 (1.03, 8.39), 0.049	3.49 (1.19, 11.14), 0.027	2.20 (0.65, 7.74), 0.21
P for trend	0.014	0.011	0.106

Note: Data are shown as ORs with 95% CIs. Model 1 was unadjusted. Model 2 was adjusted

for ethnicity and educational attainment. Model 3 was additionally adjusted for marital status, smoking status, and alcohol consumption.

Supplementary Table S6. Threshold effect analysis of the association between the Framingham Steatosis Index and breast cancer case status in the exploratory hospital-based cohort.

Outcome	OR(95%CI), P value
Fitting by standard logistic model	1.80 (1.21, 2.69), 0.0038
Fitting by two-piecewise logistic regression model	
Inflection point	-3.67
< -3.67	0.70 (0.13, 3.78), 0.6801
> -3.67	2.24 (0.16, 30.75), 0.5475
Log-likelihood ratio	0.2519

Note: The model was adjusted for ethnicity, educational attainment, marital status, smoking status, and alcohol consumption.

Supplementary figure legend

Supplementary Figure S1. Generalized additive model curves for the association between the Framingham Steatosis Index and prevalent breast cancer in NHANES 1999–2018.

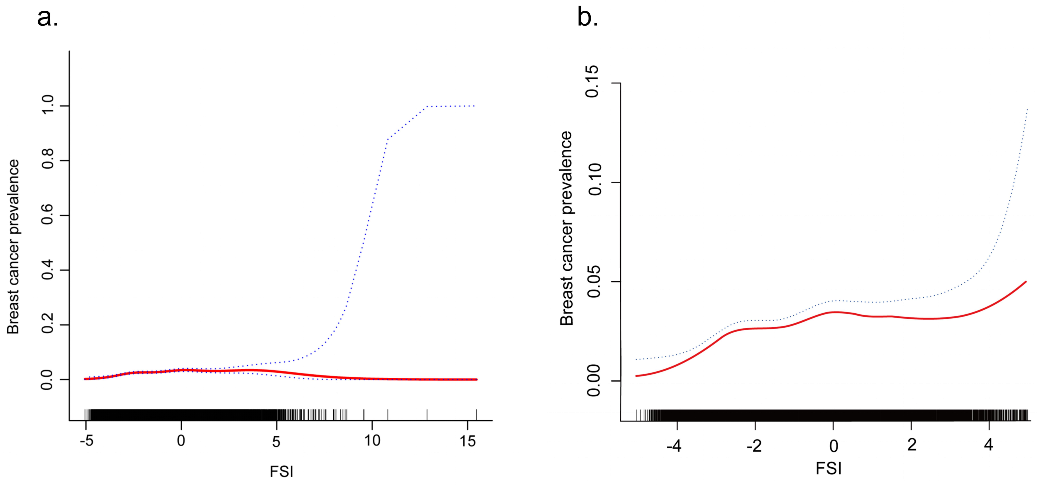

Note: The solid line indicates the smoothed fitted curve and the dashed lines indicate the 95% confidence interval. Panel (a) shows the full NHANES 1999 – 2018 cohort (N = 21,042), and panel (b) shows the cohort after exclusion of 95 extreme FSI values (N = 20,947).
